# Having a Same Type IIS Enzyme’s Restriction Site on Guide RNA Sequence Does Not Affect Golden Gate (GG) Cloning and Subsequent CRISPR/Cas Mutagenesis

**DOI:** 10.3390/ijms23094889

**Published:** 2022-04-28

**Authors:** M. Moniruzzaman, Yun Zhong, Zhifeng Huang, Guangyan Zhong

**Affiliations:** 1 Institute of Fruit Tree Research, Guangdong Academy of Agricultural Sciences, Guangzhou 510640, China; zhongyun99cn@163.com (Y.Z.); huangmo7721@163.com (Z.H.); 2 Center for Viticulture and Small Fruit Research, Florida A&M University, Tallahassee, FL 32308, USA; 3 Key Laboratory of South Subtropical Fruit Biology and Genetic Resource Utilization, Ministry of Agriculture and Rural Affairs, Guangdong Provincial Key Laboratory of Tropical and Subtropical Fruit Tree Research, Guangzhou 510640, China

**Keywords:** gRNA designing, modular cloning, restriction digestion, genome editing, base editing, plant transformation

## Abstract

Golden gate/modular cloning facilitates faster and more efficient cloning by utilizing the unique features of the type IIS restriction enzymes. However, it is known that targeted insertion of DNA fragment(s) must not include internal type IIS restriction recognition sites. In the case of cloning CRISPR constructs by using golden gate (GG) cloning, this narrows down the scope of guide RNA (gRNA) picks because the selection of a good gRNA for successful genome editing requires some obligation of fulfillment, and it is unwanted if a good gRNA candidate cannot be picked only because it has an internal type IIS restriction recognition site. In this article, we have shown that the presence of a type IIS restriction recognition site in a gRNA does not affect cloning and subsequent genome editing. After each step of GG reactions, correct insertions of gRNAs were verified by colony color and restriction digestion and were further confirmed by sequencing. Finally, the final vector containing a *Cas12a* nuclease and four gRNAs was used for *Agrobacterium*-mediated citrus cell transformation. Sequencing of PCR amplicons flanking gRNA-2 showed a substitution (C to T) mutation in transgenic plants. The knowledge derived from this study could widen the scope of GG cloning, particularly of gRNAs selection for GG-mediated cloning into CRISPR vectors.

## 1. Introduction

CRISPR/Cas-based gene editing has been considered the most promising tool for dissecting gene functions [1] and improving crops’ traits in agriculture [2,3]. The technique uses Cas proteins to make desired nucleotide changes on target genes under the guidance of a short RNA sequence called guide RNA (gRNA). In particular, the editing specificity and successfulness are largely determined by the gRNAs picked. Several factors must be considered when picking a “good” gRNA sequence, i.e., an appropriate protospacer adjacent motif (PAM), GC content, position in the gene to be edited (which should be in exons of the target gene), presence of a restriction site, and secondary structures among other things [4,5]. For confirmation of gene function aberration mutation, more than one gRNA targeting the same gene should be used to increase the chances of editing. Several gRNAs are also needed for multiplex genome editing. Therefore, techniques that facilitate efficient cloning and expression of multiple gRNAs are preferred. The modular cloning (MoClo), an improved version of GG cloning, is the technique that meets those requirements [6]. MoClo, with the use of a three-step reaction by taking the advantage of type IIS restriction enzymes, offers the packing of all modules for gene editing into a single vector [7]. Logically, it is commonly advised not to use gRNAs possessing a type II enzymes’ restriction site even if the gRNAs are “perfect” [8,9]. However, the type II enzymes’ restriction sites are frequently encountered and sometimes appropriate gRNAs may be unavoidable especially in case of precise base editing (BE) where the specific base must be present at a very short window in a gRNA [10,11]. In this short article, we showed that presence of a type II enzymes’ restriction site within the gRNA sequence does not produce significant reduction in cloning and subsequent genome editing efficiency in citrus cells.

## 2. Results

**Figure 1:**
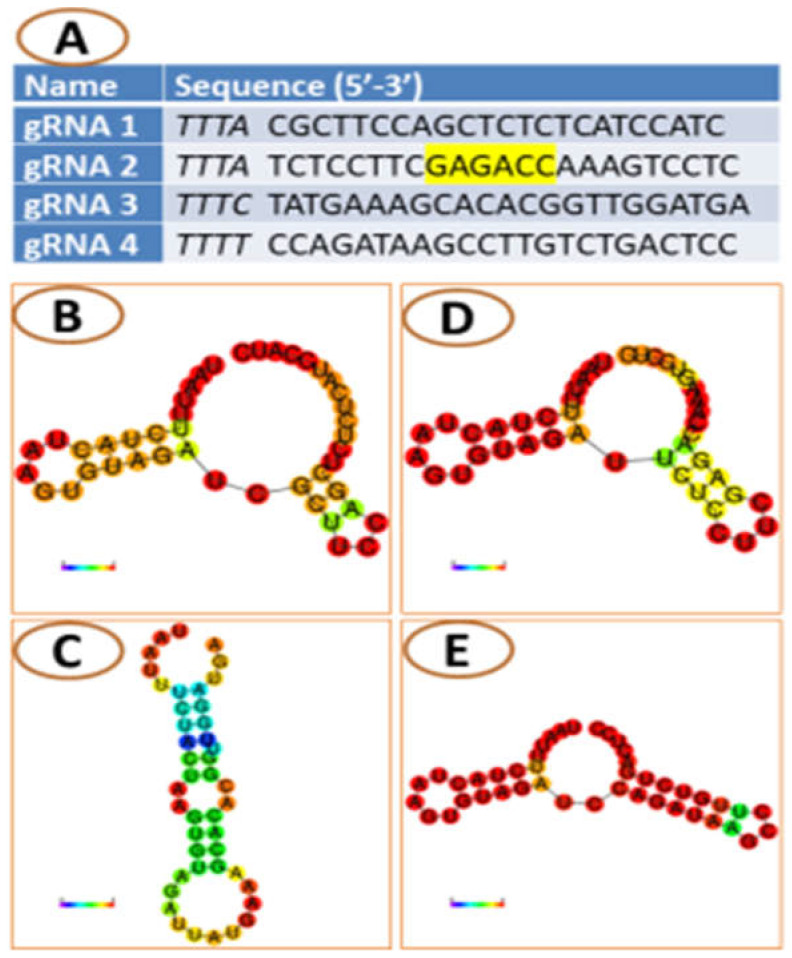
gRNAs sequence and fold structure. (**A**) 23 base gRNA for gRNA 1 to 4; PAMs are marked italic and the yellow highlight is showing the *Bsa*l restriction recognition site in the gRNA 2. (**B**–**E**) Fold structure of gRNA 1 to 4, respectively.

**Figure 2:**
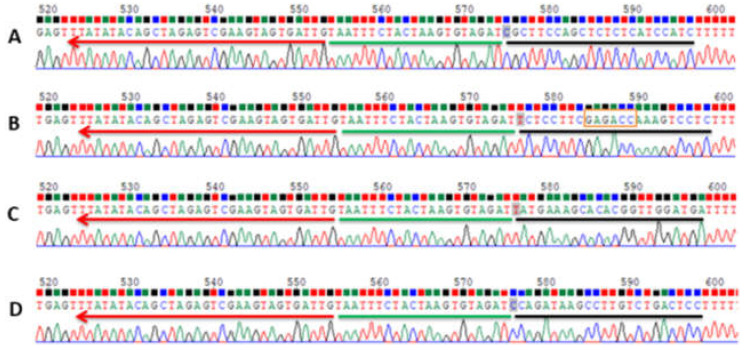
Sequencing confirmation of gRNAs insert after *Bsa*l-mediated golden gate (GG) reaction. (**A**–**D**) shows the sequences and recombination constructs of gRNA 1 to gRNA 4, respectively; black underlines represent gRNAs; green underlines represent sgRNA scaffold and red underlines represent the sequence from pH103; the red marked box in gRNA 2 shows the intact *Bsa*l recognition site.

**Figure 3:**
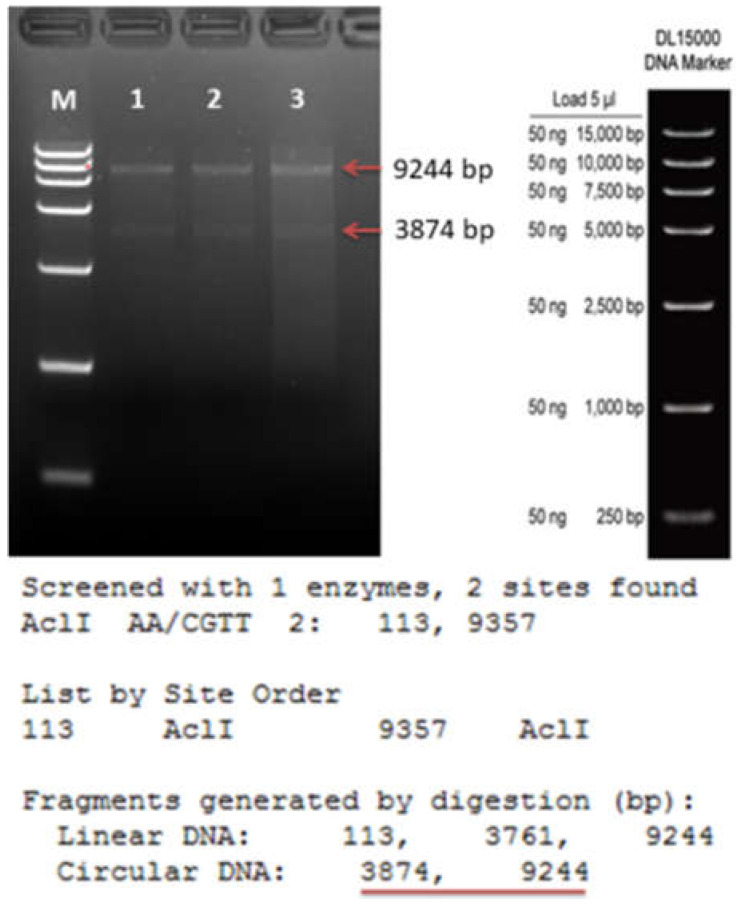
*AclI* (AA/CGTT) restriction digestion conformation of the L2 final vector. M represents marker lane and 1 to 3 represents plasmids from 3 individual colonies. Red arrows show the bands of digested fragments in gel; red underline shows the fragment length of the virtual vector.

Two exons are present in the *CsACD2* gene. Correspondingly, six and seventeen gRNAs were proposed by an online program (CRISPOR) for Exon-1 and Exon-2. Four gRNAs (guide 1 to 4; Figure 1) were picked after passing the evaluation standard of off-target cleavage, position, and secondary structure. Among the in silico selected guides, the best one was guide 2 (gRNA-2) as it is located in the first exon, not far from the start codon. The RNA folding integrity was properly maintained and, most importantly, there was no off-target cleavage. However, guide 2 suffered a drawback, as it contained a *Bsa*1 restriction site, 5′(N)5GAGACC….3′ (Figure 1), that is one of the two type IIS restriction enzymes (*Bpil* and *Bsa*1) used for the modular cloning in this experiment. Despite this drawback, we set out to proceed with guide 2 to test the usability and efficiency of guide 2 in cloning and gene editing. After went through all previous cloning (Level 0 and Level 1), finally, guide 2, was cloned into a Level 2 CRISPR vector (pICSL4723) along with the remaining three guides and other expression cascades that are required for CRISPR/Cas function. Plenty of white colonies grew on agar plates with IPTG+X-Gal when transformed bacteria (*E. coli*) were plated at all levels of cloning. Three well-distanced white colonies were picked and individually cultured in liquid LB media containing appropriate antibiotic(s). Plasmids from all levels of cloning were extracted and sequenced by using appropriate primers (Supplement S3). The results showed that, like the other three gRNAs, the guide 2 insert was present in the L0 and L1 plasmids in correct orientation (Figure 2). Moreover, the final L2 vectors (13,118 bp, Supplement S4) contained all of the four guides, a Cas12a expression cassette and a Basta selection marker. The final plasmid (L2 vector) was verified by an *AclI* (AA/CGTT) restriction digest, which generated two fragments (3874 bp and 9244 bp Figure 3).

**Figure 4:**
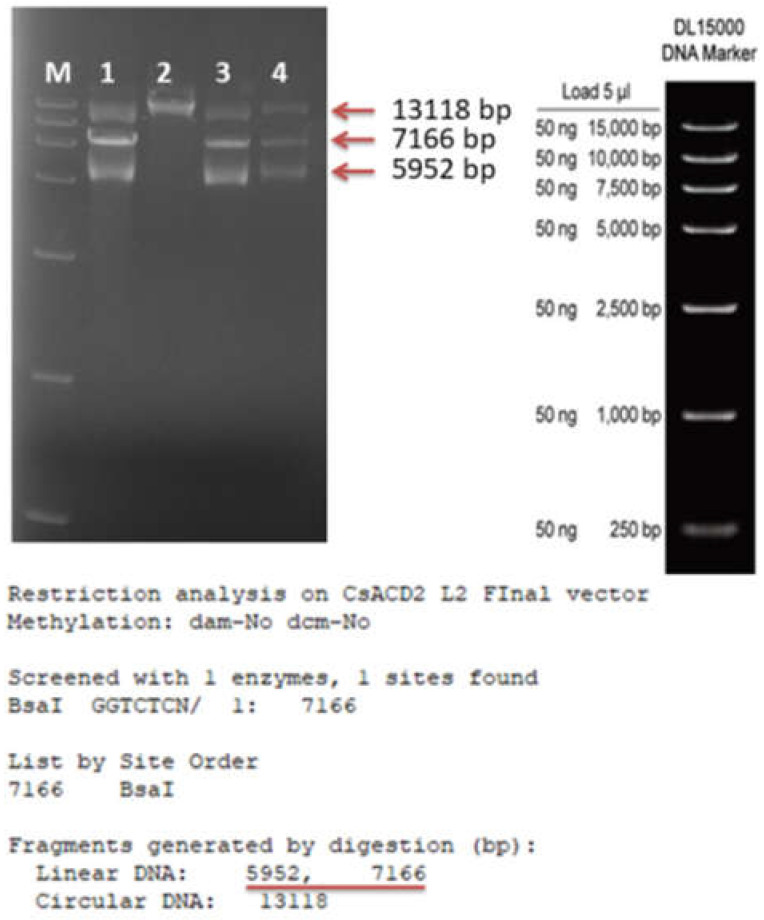
*Bas*l restriction digest conformation of L2 final vector. M represents marker lane and 1 to 4 represents plasmids from 4 individual colonies. Red arrows show the bands for intact plasmid and digested fragments in gel; red underline shows the fragment length of the virtual vector.

For further conformation, we checked (in silico) the L2 virtual vector to cut with *Bsa*1 and found the cut will produce two fragments (5952 bp and 7166 bp). After digestion treatment and gel electrophoresis, the expected bands (5952 bp and 7166 bp) were shown along with a non-cut plasmid band (−13,118 bp) (Figure 4), meaning that all the desired units are present in the plasmid and the treatment might not cleave all the available plasmids to digest. Furthermore, we performed a GG reaction (as was done previously) by using a *Bsa*1 enzyme where the L2 vector was used as substrate. After the GG reaction, the aliquot was transformed to *E. coli* and cultured on a kanamycin- supplemented LB agar plate with IPTG+X-Gal to check whether, it can produce the white colonies. On the following day, white colonies were observed, meaning that some of the plasmids were intact after the GG reaction. For further conformation, we recovered the plasmids from three of these white colonies for *Bsa*1 digestion and gel electrophoresis. After restriction-digestion and gel run, we found similar bands as non-cut plasmid (−13,118 bp), and cut plasmid (5952 bp and 7166 bp) (Figure 4), confirming that the plasmid contained all the desired units that are being incorporated and that some of the plasmids were intact after GG reaction. This finding may be because the restriction enzyme was not able to cut all the available plasmids or re-ligate the cut ends because of the available corresponding overhang sequence.

**Figure 5:**
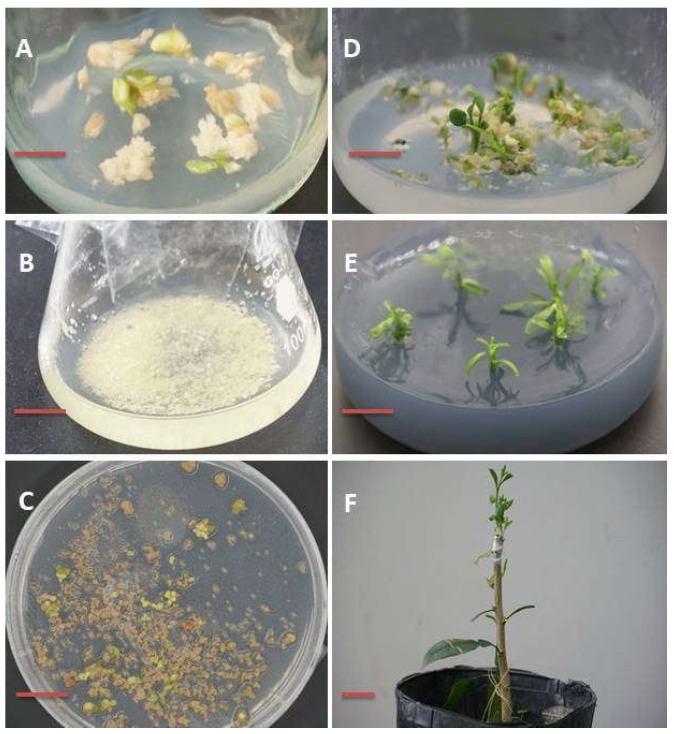
Embryonic callus induction, suspension cell culture establishment, and transgenic plant regeneration of sweet orange (*Citrus sinensis*) cultivar. (**A**) Embryonic callus induction from 8 weeks old ovule. (**B**) Suspension cell culture establishment. (**C**) Callus formation and embryo germination from putative transformed cell on *basta* supplement medium. (**D**) Axis elongation of germinated embryos. (**E**) Plants on rooting medium. (**F**) In vitro shoot grafted on rootstock plant. The bars represent 1 cm.

**Figure 6:**
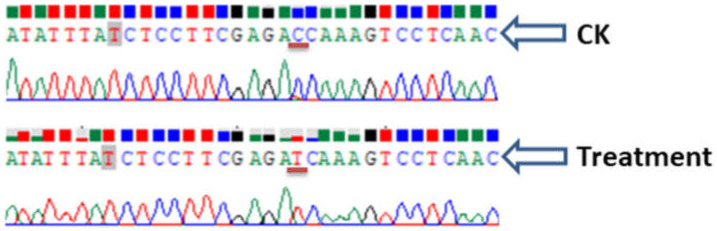
Sequencing confirmation of mutation in transgenic plant. Red underline represents the substitution mutation.

Citrus suspension cells (*Citrus sinensis*) were transformed with the vector and putative transformed cells were selected on Basta-supplemented (20 mg/L) media [3]. Basta- survived cells were then in vitro regenerated to recover genome-edited transgenic plants (Figure 5). PCR amplification and subsequent sequencing clearly showed that there was a substitution mutation (C to T) in case of gRNA-2 (Figure 6).

## 3. Discussion

The type IIS restriction enzyme mediated GG cloning is superior over traditional cloning techniques as the entire cloning steps (digestion and ligation) can be carried out in one reaction with the use of a single type IIS restriction enzyme [6]. Because the recognition site is designed to be removed in the cloning step, the method allows cloning of multiple inserts into one vector by a single reaction. It is therefore a faster and more efficient cloning method. CRISPR technology combined with GG modular cloning approach could become a very powerful strategy for understanding gene functions as well as for manipulating gene sequences, and, hence, a better approach to crop genetic improvement [7].

The major disadvantage of the modular cloning method is that the “type IIS sites can be found throughout DNA sequences and it was assumed that the type IIS site must not be present within the fragments seeking to be assembled” [8,9]. We have shown that the presence of the type IIS site does not affect cloning efficacy significantly because the vector with guide 2 insert was successfully obtained in this study. There were two probable scenarios responsible for what had happened. The first one is involved with the unit base cleavage [12] feature of restriction enzyme and the shortened digestion time of the given GG reaction [7]. We set the GG reaction as (37 °C for 3 min and 16 °C for 4 min) × 50 cycles, 50 °C for 5 min and 80 °C for 5 min then store at 4 °C. The enzyme showed activity and cut the desired DNA at 37 °C. But at one cycle, the treatment time was too short (3 min) to cut all the restriction sites in the given DNA, though there was still enough cut to release the very specific fusion sites that allowed the insertion and ligation of the desire DNA fragments at the next 16 °C incubation. For example, the restriction enzyme cut some of the available plasmids to release the specific fusion sites, at the same time there were some intact gRNA 2 that have the appropriate overhang for ligation with the cut plasmids; as a result they (gRNA 2) were correctly cloned into the plasmids. And the second probable scenario was that the cut within gRNA 2 was also made by the enzyme but the same cut was re-ligated again in the following ligation reaction as there was no other appropriate overhang sequence to ligate. Any of these or both scenarios could happen at the same time for making the appropriate cloning.

We further checked the mutation created by gRNA 2. We found some putative plant cells were grown on Basta-supplemented media (Figure 5C), meaning that the vector was successfully transformed into the cells, because the vector contained *Basta* selection marker gene for plant selection [3]. A substitution mutation at the 13th base (C to T) of gRNA 2 was produced (Figure 6), which confirmed that the CRISPR vector was functional. CRISPR/Cas-mediated genome editing has been reported in *Citrus*; different types, including deletions, nucleotide substitutions, and insertion have been reported [13,14]. In our experiment, we found a base substitution that is consistent with previous reports.

In conclusion, we could tell that having a type IIS restriction site at guide RNA will not significantly affect GG cloning and subsequent genome edition. Our discovery will broaden the scope of GG cloning and gRNA selection for CRISPR experiment.

## 4. Materials and Methods

*CsACD2* was reported to be a susceptible gene in the pathogenesis of citrus Huaglongbing [15]. The gene was chosen as the target of our gene editing experiment. Its coding sequence (*CsACD2*; *Citrus sinensis* GCF_000317415.1) was retrieved from the NCBI databank and confirmed by sequencing of PCR amplicons (primer MoACD2) from sweet orange (*Citrus sinensis*) genomic DNA. The gRNAs (Cas12a PAM (TTTV) and 23 bp gRNA) were designed for targeting the exons of *CsACD2* by using CRISPOR [16], the online gRNA designing tool, with default settings. Given gRNAs were evaluated individually for positions within the target gene, restriction site presence, and secondary structures. Restriction site and secondary structure inspections were performed by using SnapGene (Version: 5.3) and RNAfold (http://rna.tbi.univie.ac.at/cgi-bin/RNAWebSuite/RNAfold.cgi), respectively (Last access 10 November 2020).

The modular cloning approach was used to efficiently clone and pack all required modules into a level 2 backbone, pICSL4723, as described in a previous article [7]. The plasmids, pFH103, pFH35, pEPOR1CB0013, and pICSL11017, were respectively used for crRNA backbone of LbCas12a, AtU6-26 promoter, *LbCas12a* expression cassette and *Basta* expression cassette. Whereas pICH47751, pICH47761, pICH47772, pICH47781 were used as an empty backbone for gRNA expression cascade of guide gRNA- 1 to gRNA- 4 respectively. Plasmid pICH41822 was used as end-linker (for four guides). All required modules were cloned by three-step GG reactions by using either *Bpi*l or *Bsa*1 restriction enzyme (detailed cloning procedure are given in Supplement S1.1 and S1.2). The expression of *Basta* and *Cas12a* were controlled by 35S promoter and the expression of individual gRNAs was driven by *Arabidopsis* U6-26 Pol III promoter (Supplement S2). Two microliter (2 µL) of each cloning reaction was used to transform 50 µL of competent *E. coli* (DH10B strain) and then subjected to selection on LB agar media supplemented with appropriate antibiotic(s) and/or X-Gal/IPTG. Colonies from the L0/L1 GG reactions and the L2 reaction were primarily verified by white/blue and white/red screening, respectively. Three positive (white) colonies were picked from each plate after each GG reactions and examined for inserts by PCR plus sequencing (by using primer FH32 for the first, Lvl1_R(0230) for the second and MOg2 for the third reaction; all primers sequences are given Supplement S3). The final L2 vector was further confirmed by restriction digestion by using *Acl*I and subsequent gel electrophoresis (the full sequence of final L2 vector is given in Supplement S4). The L2 vector was further treated with *Bsa*1 enzyme. Then electrophoresis was performed to check whether it leaves any intact plasmid or not. Moreover, the vector (L2) was subjected to a GG reaction by using a *Bsa*1 enzyme to check whether it could produce an intact L2 vector again.

Plant transformation and regeneration of transgenic plants were conducted as described in our recent article [3]. Briefly, the L2 final vector was introduced into EHA105 *Agrobacterium* and allowed to infect the citrus (*Citrus sinensis*) cell suspension. Putative transformed cells were in vitro regenerated. Leaf samples from the regenerated transformants were analyzed for transgenes and for CRISPR mutations. Specifically, DNA sequences were amplified from the transformants and the amplicons were sequenced to identify mutations by using primer MoACD2. Sequencing histograms were analysed by using Chromas 2.6.6 and SnapGene 5.3.

## Data Availability

Not applicable.

## Supplementary Materials

The following supporting information can be downloaded at: https://www.mdpi.com/article/10.3390/ijms23094889/s1.
