# Supplementary material for: Having a Same Type IIS Enzyme’s Restriction Site on Guide RNA Sequence Does Not Affect Golden Gate (GG) Cloning and Subsequent CRISPR/Cas Mutagenesis
Source: Int J Mol Sci. 2022 Apr 28;23(9):4889. doi: 10.3390/ijms23094889 (PMC9101711; doi:10.3390/ijms23094889)
Supplement: Supplementary file 1 [file ijms-23-04889-s001.zip › ijms-1672378-supplementary.pdf]

Online Supplement

Supplement S1.1: Golden Gate (GG) reactions

Step 1 (First GG reaction): Integration of target site into modular vector system

GG reaction 1a for 1st gRNA

1 µL	pFH103 (adjusted to 100 ng/µL)
1 µL	AGAT $(N)_{23}$ Overhangs oligos (Forward primer -10 µM)
1 µL	AAAA $(N)_{23\ reverse\ complement}$ Overhangs oligos (Reverse primer -10 µM)
1.5 µL	10x T4 DNA Ligase buffer (New England Biolabs)
1.5 µL	BSA (1 mg/mL, New England Biolabs)
1 µL	BpiI (1 mg/mL, New England Biolabs)
1 µL	T4 DNA Ligase (400000 U/µL, New England Biolabs)
7 µL	ddH_2O (adjust final volume to 15 µL)

GG reaction ***1b, 1c and 1d for 2nd gRNA, 3rd gRNA and 4th gRNA*** *respectively. Here the only difference will be the 20/23 bp gRNA sequence according to our choice***.**

Step 2 (Second GG reaction): Assembly of transcriptional units for gRNA expression and nuclease expression

GG reaction 2a (for 1st gRNA, Position 3 at L2 construct)

1 µL	pICH47751 (adjusted to 100 ng/µL)
1 µL	pFH35 (adjusted to 100 ng/µL)
1 µL	pFH103a (adjusted to 100 ng/µL), I put "a" for 1st gRNA
1.5 µL	10x T4 DNA Ligase buffer (New England Biolabs)
1.5 µL	BSA (1 mg/mL, New England Biolabs)
1 µL	Bsa1 (1 mg/mL, New England Biolabs)
1 µL	T4 DNA Ligase (400000 U/µL, New England Biolabs)
7 µL	ddH_2O (adjust final volume to 15 µL)

GG reaction 2b (for 2nd gRNA, Position 4 at L2 construct)

1 µL	pICH47761 (adjusted to 100 ng/µL)
1 µL	pFH35 (adjusted to 100 ng/µL)
1 µL	pFH103b (adjusted to 100 ng/µL), I put "b" for 2nd gRNA
1.5 µL	10x T4 DNA Ligase buffer (New England Biolabs)
1.5 µL	BSA (1 mg/mL, New England Biolabs)
1 µL	Bsa1 (1 mg/mL, New England Biolabs)
1 µL	T4 DNA Ligase (400000 U/µL, New England Biolabs)
7 µL	ddH_2O (adjust final volume to 15 µL)

GG reaction 2c (for 3rd gRNA, Position 5 at L2 construct)

1 µL	pICH47772 (adjusted to 100 ng/µL)
1 µL	pFH35 (adjusted to 100 ng/µL)
1 µL	pFH103c (adjusted to 100 ng/µL), I put "c" for 3rd gRNA
1.5 µL	10x T4 DNA Ligase buffer (New England Biolabs)

1.5 µL	BSA (1 mg/mL, New England Biolabs)
1 µL	Bsa1 (1 mg/mL, New England Biolabs)
1 µL	T4 DNA Ligase (400000 U/µL, New England Biolabs)
7 µL	ddH_2O (adjust final volume to 15 µL)

GG reaction 2d (for 4th gRNA, Position 6 at L2 construct)

1 µL	pICH47781 (adjusted to 100 ng/µL)
1 µL	pFH35 (adjusted to 100 ng/µL)
1 µL	pFH103d (adjusted to 100 ng/µL), I put "d" for 4th gRNA
1.5 µL	10x T4 DNA Ligase buffer (New England Biolabs)
1.5 µL	BSA (1 mg/mL, New England Biolabs)
1 µL	Bsa1 (1 mg/mL, New England Biolabs)
1 µL	T4 DNA Ligase (400000 U/µL, New England Biolabs)
7 µL	ddH_2O (adjust final volume to 15 µL)

Step 3: (Third GG reaction) Assembly of multigene construct

1 µL	L2 backbone -pICSL4723 (adjusted to 100 ng/µL)
1 µL	BAR marker cascade, pICSL11017 (adjusted to 100 ng/µL)
1 µL	LbCas 12a nuclease cascade, pEPOR1CB0013 (adjusted to 100 ng/µL)
1 µL	End-linker - pICH41822, for 4 gRNAs
1 µL	pICH47751 for 1st gRNA
1 µL	pICH47761 for 2nd gRNA
1 µL	pICH47772 for 3rd gRNA
1 µL	pICH47781 for 4th gRNA
1.5 µL	10x T4 DNA Ligase buffer (New England Biolabs)
1.5 µL	BSA (1 mg/mL, New England Biolabs)
1 µL	BpiI (1 mg/mL, New England Biolabs)
1 µL	T4 DNA Ligase (400000 U/µL, New England Biolabs)
2 µL	ddH_2O (adjust final volume to 15 µL)

Supplement S1.2: GG reaction condition

37 ˚C	20 s	
37 ˚C	3 min	} 50x
16 ˚C	4 min	
50 ˚C	5 min	
80 ˚C	5 min	
4 ˚C	store	

Supplement S2: Configuration of vector elements between LB and RB of Ti plasmid

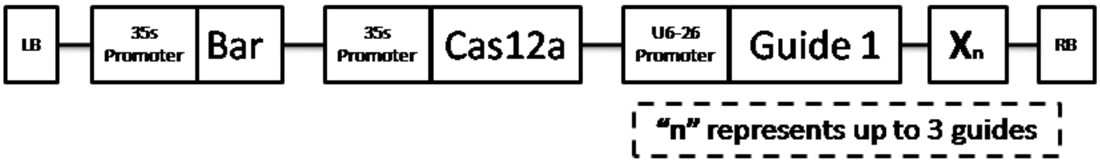

Supplement S3: Primers used in this study

Name	Type	Sequence (5'-3')
FH32	Sequencing	GCAATGTAACATCAGAGATTTTGA
Lvl1_R(0230)	Sequencing	CTGGTGGCAGGATATATTGTGGTG
MOg2	Sequencing	TTACGAATTCCCATGGGGA
MoACD2	PCR and Sequencing	Forward: AAACTTTCACGGCGAAGC<br>Reverse: CACACTGATTTAGCCAGATTCC

Supplement S4: DNA sequences of CRISPR/Cas12a final vector

```
>gaacactctgtgccgaattcggatccggagcggagaattaagggagtcacgttatgacccccgc
cgatgacgcgggacaagccgttttacgtttggaactgacagaaccgcaacgttgaaggagccact
cagccgcgggtttctggagtttaatgagctaagcacatacgtcagaaaccattattgcgcgttca
aaagtcgcctaaggtcactatcagctagcaaatatttcttgtcaaaatgctccactgacgttcca
taaattcccctcggtatccaattagagtctcatattcactctcaatccaaataatctgcaccgga
tctggatcgtttcgcatgtctcctgaaagaaggcctgctgatattagaagggctactgaggctga
tatgcctgctgtgtgcactattgtgaatcattacattgagacttctactgtgaatttcaggactg
agcctcaagaaccacaagagtggactgatgatcttgtgaggcttagagagaggtatccttggctt
gttgctgaagtggatggtgaagtggctggtattgcttatgctggtccttggaaggctaggaatgc
ttacgattggactgctgagtctactgtttacgtgtctcctaggcatcaaagaactggtcttggat
ctactctttacactcatcttttgaagtctttggaggctcagggtttcaagtctgtggttgctgtg
attggacttcctaatgatccttctgtgaggatgcatgaggctcttggttatgctcctaggggaat
gcttagagctgctggtttcaaacatggaaattggcatgatgtgggattctggcagcttgatttct
ctcttcctgttcctcctagacctgttcttcctgtgactgagatttgagcgggactctggatctag
agtcaagcagatcgttcaaacatttggcaataaagtttcttaagattgaatcctgttgccggtct
tgcgatgattatcatataatttctgttgaattacgttaagcatgtaataattaacatgtaatgca
tgacgttatttatgagatgggtttttatgattagagtcccgcaattatacatttaatacgcgata
gaaaacaaaatatagcgcgcaaactaggataaattatcgcgcgcggtgtcatctatgttactaga
tcgacgcttgctgaattggagcgctgcaagaattcaagcttggaggtcaacatggtggagcacga
cactctggtctactccaaaaatgtcaaagatacagtctcagaagatcaaagggctattgagactt
ttcaacaaaggataatttcgggaaacctcctcggattccattgcccagctatctgtcacttcatc
gaaaggacagtagaaaaggaaggtggctcctacaaatgccatcattgcgataaaggaaaggctat
cattcaagatctctctgccgacagtggtcccaaagatggacccccacccacgaggagcatcgtgg
aaaaagaagaggttccaaccacgtctacaaagcaagtggattgatgtgataacatggtggagcac
gacactctggtctactccaaaaatgtcaaagatacagtctcagaagatcaaagggctattgagac
ttttcaacaaaggataatttcgggaaacctcctcggattccattgcccagctatctgtcacttca
tcgaaaggacagtagaaaaggaaggtggctcctacaaatgccatcattgcgataaaggaaaggct
```

atcattcaagatctctctgccgacagtggtcccaaagatggacccccacccacgaggagcatcgt
ggaaaaagaagaggttccaaccacgtctacaaagcaagtggattgatgtgacatctccactgacg
taagggatgacgcacaatcccactatccttcgcaagacccttcctctatataaggaagttcattt
catttggagaggacacgctcgagtataagagctcatttttacaacaattaccaacaacaacaaac
aacaaacaacattacaattacatttacaattatcgatacaatgagcaagctggagaagtttacaa
actgctactccctgtctaagaccctgaggttcaaggccatccctgtgggcaagacccaggagaac
atcgacaataagcggctgctggtggaggacgagaagagagccgaggattataagggcgtgaagaa
gctgctggatcgctactatctgtcttttatcaacgacgtgctgcacagcatcaagctgaagaatc
tgaacaattacatcagcctgttccggaagaaaaccagaaccgagaaggagaataaggagctggag
aacctggagatcaatctgcggaaggagatcgccaaggccttcaagggcaacgagggctacaagtc
cctgtttaagaaggatatcatcgagacaatcctgccagagttcctggacgataaggacgagatcg
ccctggtgaacagcttcaatggctttaccacagccttcaccggcttctttgataacagagagaat
atgttttccgaggaggccaagagcacatccatcgccttcaggtgtatcaacgagaatctgacccg
ctacatctctaatatggacatcttcgagaaggtggacgccatctttgataagcacgaggtgcagg
agatcaaggagaagatcctgaacagcgactatgatgtggaggatttctttgagggcgagttcttt
aactttgtgctgacacaggagggcatcgacgtgtataacgccatcatcggcggcttcgtgaccga
gagcggcgagaagatcaagggcctgaacgagtacatcaacctgtataatcagaaaaccaagcaga
agctgcctaagtttaagccactgtataagcaggtgctgagcgatcgggagtctctgagcttctac
ggcgagggctatacatccgatgaggaggtgctggaggtgtttagaaacaccctgaacaagaacag
cgagatcttcagctccatcaagaagctggagaagctgttcaagaattttgacgagtactctagcg
ccggcatctttgtgaagaacggccccgccatcagcacaatctccaaggatatcttcggcgagtgg
aacgtgatccgggacaagtggaatgccgagtatgacgatatccacctgaagaagaaggccgtggt
gaccgagaagtacgaggacgatcggagaaagtccttcaagaagatcggctcctttctctggagc
agctgcaggagtacgccgacgccgatctgtctgtggtggagaagctgaaggagatcatcatccag
aaggtggatgagatctacaaggtgtatggctcctctgagaagctgttcgacgccgattttgtgct
ggagaagagcctgaagaagaacgacgccgtggtggccatcatgaaggacctgctggattctgtga
agagcttcgagaattacatcaaggccttctttggcgagggcaaggagacaaacagggacgagtcc
ttctatggcgattttgtgctggcctacgacatcctgctgaaggtggaccacatctacgatgccat
ccgcaattatgtgacccagaagccctactctaaggataagttcaagctgtattttcagaaccctc
agttcatgggcggctgggacaaggataaggagacagactatcgggccaccatcctgagatacggc
tccaagtactatctggccatcatggataagaagtacgccaagtgcctgcagaagatcgacaagga
cgatgtgaacggcaattacgagaagatcaactataagctgctgcccggccctaataagatgctgc
caaaggtgttcttttctaagaagtggatggcctactataaccccagcgaggacatccagaagatc
tacaagaatggcacattcaagaagggcgatatgtttaacctgaatgactgtcacaagctgatcga
cttctttaaggatagcatctcccggtatccaaagtggtccaatgcctacgatttcaacttttctg
agacagagaagtataaggacatcgccggcttttacagagaggtggaggagcagggctataaggtg
agcttcgagtctgccagcaagaaggaggtggataagctggtggaggagggcaagctgtatatgtt
ccagatctataacaaggacttttccgataagtctcacggcacacccaatctgcacaccatgtact
tcaagctgctgtttgacgagaacaatcacggacagatcaggctgagcggaggagcagagctgttc
atgaggcgcgcctccctgaagaaggaggagctggtggtgcacccagccaactcccctatcgccaa
caagaatccagataatcccaagaaaaccacaaccctgtcctacgacgtgtataaggataagaggt
tttctgaggaccagtacgagctgcacatcccaatcgccatcaataagtgccccaagaacatcttc
aagatcaatacagaggtgcgcgtgctgctgaagcacgacgataacccctatgtgatcggcatcga
taggggcgagcgcaatctgctgtatatcgtggtggtggacggcaagggcaacatcgtggagcagt

attccctgaacgagatcatcaacaacttcaacggcatcaggatcaagacagattaccactctctg
ctggacaagaaggagaaggagaggttcgaggcccgccagaactggacctccatcgagaatatcaa
ggagctgaaggccggctatatctctcaggtggtgcacaagatctgcgagctggtggagaagtacg
atgccgtgatcgccctggaggacctgaactctggctttaagaatagccgcgtgaaggtggagaag
caggtgtatcagaagttcgagaagatgctgatcgataagctgaactacatggtggacaagaagtc
taatccttgtgcaacaggcggcgccctgaagggctatcagatcaccaataagttcgagagcttta
agtccatgtctacccagaacggcttcatcttttacatccctgcctggctgacatccaagatcgat
ccatctaccggctttgtgaacctgctgaaaaccaagtataccagcatcgccgattccaagaagtt
catcagctcctttgacaggatcatgtacgtgcccgaggaggatctgttcgagtttgccctggact
ataagaacttctctcgcacagacgccgattacatcaagaagtggaagctgtactcctacggcaac
cggatcagaatcttccggaatcctaagaagaacaacgtgttcgactgggaggaggtgtgcctgac
cagcgcctataaggagctgttcaacaagtacggcatcaattatcagcagggcgatatcagagccc
tgctgtgcgagcagtccgacaaggccttctactctagctttatggccctgatgagcctgatgctg
cagatgcggaacagcatcacaggccgcaccgacgtggattttctgatcagccctgtgaagaactc
cgacggcatcttctacgatagccggaactatgaggcccaggagaatgccatcctgccaaagaacg
ccgacgccaatggcgcctataacatcgccagaaaggtgctgtgggccatcggccagttcaagaag
gccgaggacgagaagctggataaggtgaagatcgccatctctaacaaggagtggctggagtacgc
ccagaccagcgtgaagcacaaaaggccggcggccacgaaaaaggccggccaggcaaaaaagaaaa
agggatcctacccatacgatgttccagattacgcttatccctacgacgtgcctgattatgcatac
ccatatgatgtccccgactatgcctaagcttgtcaagcagatcgttcaaacatttggcaataaag
tttcttaagattgaatcctgttgccggtcttgcgatgattatcatataatttctgttgaattacg
ttaagcatgtaataattaacatgtaatgcatgacgttatttatgagatgggtttttatgattaga
gtcccgcaattatacatttaatacgcgatagaaaacaaaatatagcgcgcaaactaggataaatt
atcgcgcgcggtgtcatctatgttactagatcgacgctactagaattcgagctcggagaagcttc
gttgaacaacggaaactcgacttgccttccgcacaatacatcatttcttcttagctttttttctt
cttcttcgttcatacagttttttttttgtttatcagcttacatttcttgaaccgtagctttcgtt
ttcttctttttaactttccattcggagtttttgtatcttgtttcatagtttgtcccaggattaga
atgattaggcatcgaaccttcaagaatttgattgaataaaacatcttcattcttaagatatgaag
ataatcttcaaaaggcccctgggaatctgaaagaagagaagcaggcccatttatatgggaaagaa
caatagtatttcttatataggcccatttaagttgaaaacaatcttcaaaagtcccacatcgctta
gataagaaaacgaagctgagtttatatacagctagagtcgaagtagtgattgtaatttctactaa
gtgtagatcgcttccagctctctcatccatctttttttttcgctttacgaattcccatggggaga
agcttcgttgaacaacggaaactcgacttgccttccgcacaatacatcatttcttcttagctttt
tttcttcttcttcgttcatacagttttttttttgtttatcagcttacatttcttgaaccgtagct
ttcgttttTATGAAAGCACACGGTTGGATGActtctttttaactttccattcggagtttttgtat
cttgtttcatagtttgtcccaggattagaatgattaggcatcgaaccttcaagaatttgattgaa
taaaacatcttcattcttaagatatgaagataatcttcaaaaggcccctgggaatctgaaagaag
agaagcaggcccatttatatgggaaagaacaatagtatttcttatataggcccatttaagttgaa
aacaatcttcaaaagtcccacatcgcttagataagaaaacgaagctgagtttatatacagctaga
gtcgaagtagtgattgtaatttctactaagtgtagattctccttcgagaccaaagtcctcttttt
ttttcgctcagagaattcgcatgcggagaagcttcgttgaacaacggaaactcgacttgccttcc
gcacaatacatcatttcttcttagctttttttcttcttcttcgttcatacagtttttttttgttt
atcagcttacattttcttgaaccgtagctttcgttttcttctttttaactttccattcggagttt
ttgtatcttgtttcatagtttgtcccaggattagaatgattaggcatcgaaccttcaagaatttg

attgaataaacatcttcattcttaagatatgaagataatcttcaaaaggcccctgggaatctga
aagaagagaagcaggcccatttatatgggaaagaacaatagtatttcttatataggcccatttaa
gttgaaaacaatcttcaaaagtcccacatcgcttagataagaaaacgaagctgagtttatataca
gctagagtcgaagtagtgattgtaatttctactaagtgtagattatgaaagcacacggttggatg
atttttttttcgcttgtggaattcctcgagggagaagcttcgttgaacaacggaaactcgacttg
ccttccgcacaatacatcatttcttcttagcttttttcttcttcttcgttcatacagtttttt
ttgtttatcagcttacattttcttgaaccgtagctttcgttttcttcttttaactttccattcg
gagtttttgtatcttgtttcatagtttgtcccaggattagaatgattaggcatcgaaccttcaag
aatttgattgaataaacatcttcattcttaagatatgaagataatcttcaaaaggcccctggga
atctgaaagaagagaagcaggcccatttatatgggaaagaacaatagtatttcttatataggccc
atttaagttgaaaacaatcttcaaaagtcccacatcgcttagataagaaaacgaagctgagttta
tatacagctagagtcgaagtagtgattgtaatttctactaagtgtagatccagataagccttgtc
tgactcctttttttttcgctgagcgaggatgcacatgtgaccgagggacacgaagtgatccgttt
aaactatcagtgtttgacaggatatattggcgggtaaacctaagagaaaagagcgtttattagaa
taatcggatatttaaaagggcgtgaaaaggtttatccgttcgtccatttgtatgtgcatgccaac
cacagggttcccctcgggagtcagccgtgcggctgcatgaaatcctggccggtttgtctgatgcc
aagctggcggcctggccggccagcttggccgctgaagaaaccgagcgccgccgtctaaaaaggtg
atgtgtatttgagtaaaacagcttgcgtcatgcggtcgctgcgtatatgatgcgatgagtaaata
aacaaatacgcaaggggaacgcatgaaggttatcgctgtacttaaccagaaaggcgggtcaggca
agacgaccatcgcaacccatctagcccgcgccctgcaactcgccggggccgatgttctgttagtc
gattccgatccccagggcagtgcccgcgattgggcggccgtgcgggaagatcaaccgctaaccgt
tgtcggcatcgaccgcccgacgattgaccgcgacgtgaaggccatcggccggcgcgacttcgtag
tgatcgacggagcgccccaggcggcggacttggctgtgtccgcgatcaaggcagccgacttcgtg
ctgattccggtgcagccaagcccttacgacatatgggccaccgccgacctggtggagctggttaa
gcagcgcattgaggtcacggatggaaggctacaagcggcctttgtcgtgtcgcgggcgatcaaag
gcacgcgcatcggcggtgaggttgccgaggcgctggccgggtacgagctgcccattcttgagtcc
cgtatcacgcagcgcgtgagctacccaggcactgccgccgccggcacaaccgttcttgaatcaga
acccgagggcgacgctgcccgcgaggtccaggcgctggccgctgaaattaaatcaaaactcattt
gagttaatgaggtaaagagaaaatgagcaaaagcacaaacacgctaagtgccggccgtccgagcg
cacgcagcagcaaggctgcaacgttggccagcctggcagacacgccagccatgaagcgggtcaac
tttcagttgccggcggaggatcacaccaagctgaagatgtacgcggtacgccaaggcaagaccat
taccgagctgctatctgaatacatcgcgcagctaccagagtaaatgagcaaatgaataaatgagt
agatgaattttagcggctaaaggaggcggcatggaaaatcaagaacaaccaggcaccgacgccgt
ggaatgccccatgtgtggaggaacgggcggttggccaggcgtaagcggctgggttgtctgccggc
cctgcaatggcactggaacccccaagcccgaggaatcggcgtgacggtcgcaaaccatccggccc
ggtacaaatcggcgcggcgctgggtgatgacctggtggagaagttgaaggccgcgcaggccgccc
agcggcaacgcatcgaggcagaagcacgccccggtgaatcgtggcaagcggccgctgatcgaatc
cgcaaagaatcccggcaaccgccggcagccggtgcgccgtcgattaggaagccgcccaagggcga
cgagcaaccagatttttttcgttccgatgctctatgacgtgggcacccgcgatagtcgcagcatca
tggacgtggccgttttccgtctgtcgaagcgtgaccgacgagctggcgaggtgatccgctacgag
cttccagacgggcacgtagaggtttccgcagggccggccggcatggccagtgtgtgggattacga
cctggtactgatggcggtttcccatctaaccgaatccatgaaccgataccgggaagggaagggag
acaagcccggccgcgtgttccgtccacacgttgcggacgtactcaagttctgccggcgagccgat
ggcggaaagcagaaagacgacctggtagaaacctgcattcggttaaacaccacgcacgttgccat

gcagcgtacgaagaaggccaagaacggccgcctggtgacggtatccgagggtgaagccttgatta
gccgctacaagatcgtaaagagcgaaaccgggcggccggagtacatcgagatcgagctagctgat
tggatgtaccgcgagatcacagaaggcaagaacccggacgtgctgacggttcaccccgattactt
tttgatcgatcccggcatcggccgttttctctaccgcctggcacgccgcgccgcaggcaaggcag
aagccagatggttgttcaagacgatctacgaacgcagtggcagcgccggagagttcaagaagttc
tgtttcaccgtgcgcaagctgatcgggtcaaatgacctgccggagtacgatttgaaggaggaggc
ggggcaggctggcccgatcctagtcatgcgctaccgcaacctgatcgagggcgaagcatccgccg
gttcctaatgtacggagcagatgctagggcaaattgccctagcaggggaaaaaggtcgaaaaagc
ttctttcctgtggatagcacgtacattgggaacccaaagccgtacattgggaaccggaacccgta
cattgggaacccaaagccgtacattgggaaccggtcacacatgtaagtgactgatataaaagaga
aaaaaggcgatttttccgcctaaaactctttaaaacttattaaaactcttaaaacccgcctggcc
tgtgcataactgtctggccagcgcacagccgaacagctgcaaaaagcgcctacccttcggtcgct
gcgctccctacgccccgccgcttcgcgtcggcctatcgcggccgctggccgctcaaaaatggctg
gcctacggccaggcaatctaccagggcgcggacaagccgcgccgtcgccactcgaccgccggcgc
ccacatcaaggctccgagtgcgcggaacccctatttgtttatttttctaaatacattcaaatatg
tatccgctcatgagacaataaccctgataaatgcttcaataatattgaaaaaggaagagtatggc
taaaatgagaatatcaccggaattgaaaaaactgatcgaaaaataccgctgcgtaaaagatacgg
aaggaatgtctcctgctaaggtatataagctggtgggagaaaatgaaaacctatatttaaaaatg
acggacagccggtataaagggaccacctatgatgtggaacgggaaaaggacatgatgctatggct
ggaaggaaagctgcctgttccaaaggtcctgcactttgaacggcatgatggctggagcaatctgc
tcatgagtgaggccgatggcgtcctttgctcggaagagtatgaagatgaacaaagccctgaaaag
attatcgagctgtatgcggagtgcatcaggctctttcactccatcgacatatcggattgtcccta
tacgaatagcttagacagccgcttagccgaattggattacttactgaataacgatctggccgatg
tggattgcgaaaactgggaagaggacactccatttaaagatccgcgcgagctgtatgattttta
aagacggaaaagcccgaagaggaacttgtcttttcccacggcgacctgggagacagcaacatctt
tgtgaaagatggcaaagtaagtggctttattgatcttgggagaagcggcagggcggacaagtggt
atgacattgccttctgcgtccggtcgctcagggaggatatcggggaagaacagtatgtcgagcta
tttttgacttactggggatcaagcctgattgggagaaaataaaatattatattttactggatga
attgttttagctgtcagaccaagtttactcatatatactttagattgatttaaaacttcattttt
aatttaaaaggatctaggtgaagatcctttttgataatctcatgaccaaaatcccttaacgtgag
ttttcgttccactgagcgtcagaccccgtagaaaagatcaaaggatcttcttgagatcctttttt
tctgcgcgtaatctgctgcttgcaaacaaaaaaaccaccgctaccagcggtggtttgtttgccgg
atcaagagctaccaactctttttccgaaggtaactggcttcagcagagcgcagataccaaatact
gttcttctagtgtagccgtagttaggccaccacttcaagaactctgtagcaccgcctacatacct
cgctctgctaatcctgttaccagtggctgctgccagtggcgataagtcgtgtcttaccgggttgg
actcaagacgatagttaccggataaggcgcagcggtcgggctgaacggggggttcgtgcacacag
cccagcttggagcgaacgacctacaccgaactgagatacctacagcgtgagctatgagaaagcgc
cacgcttcccgaagggagaaaggcggacaggtatccggtaagcggcagggtcggaacaggagagc
gcacgagggagcttccagggggaaacgcctggtatctttatagtcctgtcgggtttcgccacctc
tgacttgagcgtcgatttttgtgatgctcgtcaggggggcggagcctatggaaaaacgccagcaa
cgcggcctttttacggttcctgctcggatctgttggaccggacagtagtcatggttgatgggctg
cctgtatcgagtggtgattttgtgccgagctgccggtcggggagctgttggctggctggtggcag
gatatattgtggtgtaaacaaattgacgcttagacaacttaataacacattgcggacgtttttaa
tgtactggggtt
